# Development of a competency model for general practitioners after standardized residency training in China by a modified Delphi method

**DOI:** 10.1186/s12875-021-01508-7

**Published:** 2021-08-26

**Authors:** Yun Wei, Feiyue Wang, Zhaolu Pan, Meirong Wang, Guanghui Jin, Yanli Liu, Xiaoqin Lu

**Affiliations:** 1 Department of General Practice, School of General Practice and Continuing Education, Capital Medical University, No. 10, Xitoutiao, You An Men Wai, Fengtai District, Beijing, 100069 China; 2 Department of General Practice, Beijing Tiantan Hospital, Capital Medical University, Beijing, China

**Keywords:** China, Competency, Delphi method, General practitioner, Training

## Abstract

**Background:**

The “5 + 3” residency training is the main stream general practitioner training program in China. However, a competency model is absent for evaluating the clinical competence attained by general practitioners after training. This study was conducted to develop a consensus set of competencies for general practitioners after standardized residency training in China.

**Methods:**

A modified Delphi process was deployed to develop the competency model, including two stages: (1) generation of an initial set of competencies derived from literature review, behavioral observation of GP–patient consultations, and critical incidents interview of GPs; (2) a 2-round, web-based Delphi survey of experts in general practice, selected using purposive sampling, to prioritize and gain consensus on the essential competencies of GPs.

**Results:**

From literature review, behavioral observation, and critical incidents interview, 46 competencies in 7 domains were identified. After two rounds of Delphi survey of 28 participants (the mean age was 47.9 [9.3] years and 64.3% were women) representing a range of health professionals (GPs, managers, and researchers), a consensus was reached on 50 competencies categorized into 7 domains.

**Conclusion:**

A consensus-based competency model for general practitioners in China has been identified which may be used to evaluate the general practitioners’ clinical competence after standardized training.

**Supplementary Information:**

The online version contains supplementary material available at 10.1186/s12875-021-01508-7.

## Background

Primary health care (PHC) plays a very crucial role in high-performing health care system. In recent years, hospital-centric health delivery system was prevailing in China, in which patients preferred to get medical services in large public tertiary hospitals rather than PHC institutions, leading to a perception of health services as “too difficult to access and too expensive” [1]. Therefore, several policies have been introduced to improve PHC system in China to provide citizens with affordable and equitable access to basic health care [2–4]. In 2019, Chinese government invested ¥215 billion to PHC institutions [5], increased by nearly eightfold from ¥27 billion in 2009 [6]. With strong support of the government, primary care network was widely developed around China. According to the statistics in 2019, there were 954,390 PHC institutions across China, with 4.53 billion PHC visits (accounting for 52.0% of the total visits) [7], increased by 58.9% in comparison with the PHC visits in 2009 [8].

General practitioners (GPs) are the first contact for patients within PHC system, who are often considered as “gatekeepers” of patients’ health in China. In 2011, there are three GP training models: (1) the “5 + 3” residency training model (5-year undergraduate medical education followed by 3-year standardized residency training), (2) the on-job training (1-year training for doctors who want to register as GP), (3) the “3 + 2” rural GP residency training (3-year junior college medical education followed by 2-year rural residency training) [9]. Trainees will register as GP upon completion of the “5 + 3” residency training or the on-job training and work in community health service institutions (CHSIs) or general practice department in hospitals. Trainees who have completed the “3 + 2” rural GP residency training will register as assistant GP and work in village clinics or township hospitals. The “5 + 3” residency training is the mainstream GP training program. There are general practice curriculums during undergraduate medical education, including didactic courses of basic theories and concepts of general practice, as well as CHSI-based learning designed to acquire the preliminary impression of PHC institutions. The 3-year standardized residency training for GP includes two stages: (1) hospital-based clinical rotation and (2) CHSI-based training [10, 11]. According to statistics in 2019, there were 365,000 GPs in China, with 2.61 GPs for per ten thousand population [6], and the goal was at least 2–3 GPs per ten thousand residents in 2020 [9, 12].

Recently, promoting professionalism has become an explicit objective in GP training. Evaluation of professional competence is a vital element of this initiative. Professional competence in medicine was defined as “the habitual and judicious use of communication, knowledge, technical skills, clinical reasoning, emotions, values, and reflection in daily practice for the benefit of the individual and community being served” by Epstein and Hundert in JAMA [13]. There were practical competency models in developed countries, such as the European Definition of General Practice/Family Medicine in Europe [14], CanMEDS-FM 2017 in Canada [15], The Family Medicine Milestone Project in the US [16], Workplace Based Assessment and Annual Review of Competence Progression guidance in the UK [17], and Competency profile of the Australian general practitioner at the point of fellowship in Australia [18].

In China, the evaluation of GPs’ abilities in the “5 + 3” residency training is mainly focused on process assessment, including case report, examination, objective structured clinical examination (OSCE) [19]. In recent years, many researchers have tried to explore the competencies required for the role of GP. However, only three concentrated on assessment of the clinical ability of GP trainees at the end of training process, none of them explored the performance and competency of GPs after training based on the workplace [20]. As competency-based training model was embraced in China, the evaluation of competency of GP after training in workplace was an important feedback of “5 + 3” residency training program. In response to this need, this research was conducted to develop a competency model used for evaluation of GPs after the “5 + 3” residency training.

## Methods

### Design

This was a study of developing competency model for general practitioners through a modified Delphi method. The Delphi method is a structured process for consensus-building among a diverse group of experts. The approach has commonly been adopted in medical research and remains today the most widely used method for selecting quality indicators in healthcare [21, 22]. The process ends when an agreement has been reached on the discussed topics. According to previous studies, two or three rounds are frequently used in the Delphi process [23, 24]. This study involved two rounds of questionnaires to an expert panel via e-mail from September to November 2020. All methods in the Delphi process were carried out in accordance with previous studies [23–25] and research guideline for the Delphi survey technique [26].

**Fig. 1:**
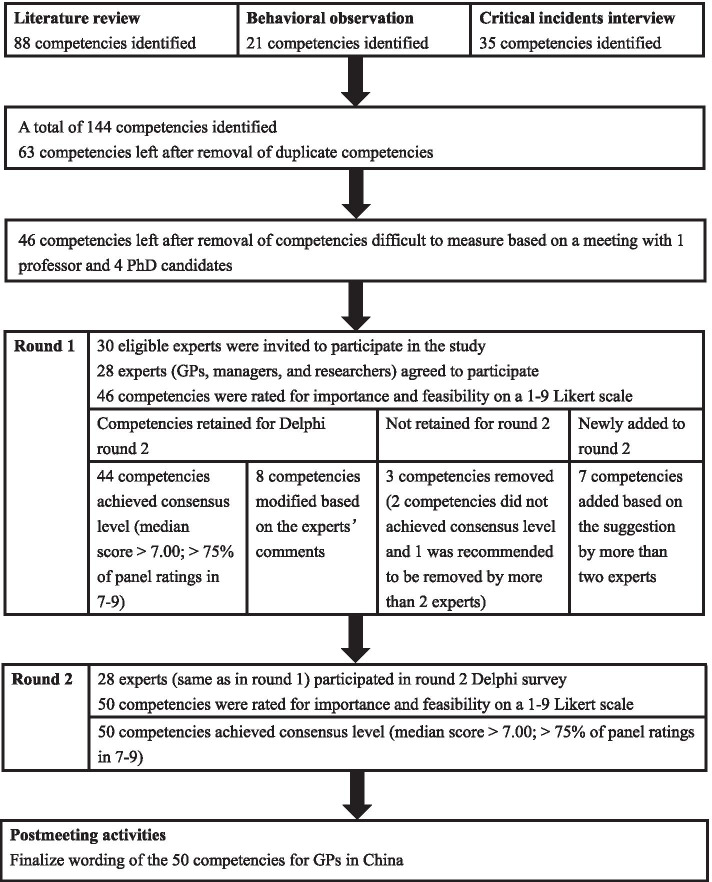
Flow diagram of the process of developing the competency model for general practitioners in China

This modified Delphi process was deployed based on two stages: (1) generating an initial set of relevant competencies derived from literature review, behavioral observation of GP–patient consultations, and critical incidents interviews of GPs; (2) conducting a 2-round, web-based Delphi survey of experts in general practice to prioritize and gain consensus on the essential competencies of GPs. Please see Fig. 1 for the process of the Delphi study.

### Participants

A list of eligible experts was initially selected considering the representativeness of potential differences in background, occupational environment and clinical practices. The experts were invited upon the following criteria: (1) working as GP, researcher, or administrative leader in general practice department; (2) having at least 5 years’ working experience in general practice; (3) knowing about the work content of GP; (4) being familiar with “5 + 3” residency training; (5) being from various geographic regions within China. The participants were asked for their willingness to take part in the study. As in previous studies, a sample of at least fifteen has been suggested and more participants can increase the variety of expertise [27, 28]. Finally, 30 eligible experts were invited and 28 experts agreed to participate in this study.

### Questionnaire preparation

Potential competencies were generated based on literature review, behavioral observations, and critical incidents interviews.

### Literature review

A preliminary list of competencies was constructed from three sources by literature review. Firstly, literature was searched in PubMed and three Chinese databases (China National Knowledge Infrastructure, Wanfang Data, VIP Chinese Periodical Services) with terms commonly used to describe GP (e.g., general practitioner, family physician, family doctor, community health worker), competency (e.g., competency, competencies, core competencies) and evaluation (e.g., evaluation, measurement, tool, indicator). A total of 37 published research papers describing domestic and foreign GPs’ competencies were identified form literature review (references of these 37 papers were shown at supplementary file 1). Secondly, 5 published competency models from international general practice organizations were also identified, including: the World Organization of Family Doctors (WONCA) [14], the College of Family Physicians of Canada (CFPC) [15], the Accreditation Council for Graduate Medical Education (ACGME) [16], the Royal College of General Practitioners (RCGP) [17], the Royal Australian College of General Practitioners (RACGP) [18]. Thirdly, 2 published policy documents of the content and requirement of GP residency training in China were reviewed [11, 29].

Potential competencies were extracted from these sources and screened by a panel of 2 reviewers (YW and FYW, Ph.D. candidates) according to the following criteria: (1) the indicators were used to measure the competency of GP; (2) the indicators were relevant to requirements of GPs’ work in China; (3) the indicators was relevant to the “5 + 3” residency training content. When there were doubts about whether an indicator should be retained, the research team would discuss together to make a decision. There were 88 competencies identified by the screening process.

### Behavioral observation

Eleven GPs from 5 community health service institutions (CHSIs) in Beijing with stable amounts of patient visits were observed as a convenience sample. Each participating GP was observed when providing medical care in general practice consultations for one workday during November 2019 to January 2020. All consecutive patients visiting the GPs on the observing workday were recruited in the study with oral agreement. During the observation, patients’ reasons for encounter (RFEs) and medical services provided by GPs were recorded. Three research assistants (YW, FYW, and ZLP, Ph.D. candidates) were trained as observers before the observation. During the observation, the observers were seated in the least intrusive corner of consulting room and will not talk to the GPs and patients. There were 21 competencies related to GPs’ work content identified by the behavioral observation process.

### Critical incidents interview

The same 11 GPs as in behavioral observation were invited, 8 GPs participate in the critical incidents interview and 3 GPs declined due to other work or family affairs. During the interview, participants were asked to describe incidents with good effect and incidents with bad effect. Questions were asked based the “STAR Principle”, which included ‘What kind of situation was it at that time?’ (Situation), ‘What was the main task you faced at that time?’ (Task), ‘In that incident, what skills did the you display?’ (Action), ‘What was the final result of this incident?’ (Result). The information from incident interview was taped, transcribed, and coded. Three researchers (YW, FYW, and ZLP, Ph.D. candidates) extracted the information about GPs’ competencies from the incident interview data respectively. When there were doubts about whether a description of competency should be retained, the research team would discuss together to make a decision. There were 35 competencies related to GPs’ work content identified by the critical incidents interview process.

A total of 144 competencies were identified by these three processes above. After deleting duplicate competencies and integrating the competencies with similar dimensions, a preliminary list of 63 potential competencies were constructed. Then, the competencies were discussed in detail one by one in a research team meeting (2 educators and 3 GPs), concentrating on whether these competencies were measurable and wording them by referring to other competency models. The competencies considered as unmeasurable by more than half of the participants were removed. After further removal and integration, 46 potential competencies were left, which were categorized into 7 domains.

### Delphi questionnaire

All 46 potential competencies were formatted into Delphi questionnaire. Importance and feasibility of the competencies were rated on a 1–9 Likert scale (1 = not important/feasible; 9 = very important/feasible). Spaces were left for experts to make comments on these existing competencies or recommend new competencies which they considered should be included in.

### Delphi survey

#### First round

The first round of Delphi survey was performed in 4 weeks from September to October 2020. Materials were sent to experts by e-mail, including first-round questionnaire, research background, and basic demographic information collection form. In the first-round questionnaire, experts were asked to rate the importance and feasibility of each competency, and give their comments.

After the first round of Delphi survey, data was collected and analyzed. The median and the distribution of scores (frequency count of answer choices), and comments were reported. For the experts’ comments, including modification, deletion and addition, we sort out and make a summary of comments expressed by at least two participants [30]. If the comments were expressed by two (or more) participants from the same professional field, further discussion was conducted in our research team.

#### Second round

The second round of Delphi survey was performed from October to November 2020, lasting 4 weeks. The second-round questionnaire was sent to experts who had completed the first-round questionnaire by e-mail. In the second-round questionnaire, the competencies which were achieved consensus level or modified based on comments in the first round were retained for Delphi round 2. New competencies were added based on the suggestion by more than two experts in the first round. Competencies were removed which did not achieved consensus level or was recommended to be removed by more than 2 experts. Along with the second-round questionnaire, graph-based report of the results of the first round was also sent to experts. Importance and feasibility of each competency were rated using the same 1–9 Likert scale as in the first round.

#### Consensus

There is no definite consensus criteria for the Delphi study [31]. In this study a consensus was reached based on two selection criteria: median score greater than seven on a nine-point scale and at least 75% of panel ratings in the top tertile (7–9) for importance and feasibility.

### Statistical analysis

Descriptive analysis was used to describe the characteristics of participates and results. Means [with standard deviation (SD)] were used to report continuous variables, while frequencies (%) were used to report categorical variables. The Data management and analysis were performed using Statistical Package for Social Science (SPSS), version 22.0.

## Results

### Panel characteristics in Delphi survey

All of the 28 experts participated in both two rounds of Delphi survey. Among them, 21 experts were from Beijing, 2 experts were from Shanghai, and other 5 experts were from 5 provinces of China (Hainan, Zhejiang, Anhui, Hebei, Inner Mongolia). There were more female participants (64.3%) in this panel, and the mean age of the experts was 47.9 years (standard deviation: 9.3 years). Nearly one third of the participants were GPs in CHSI, 39.3% were GPs in hospital, 10.7% were researchers in primary care, and 17.9% were leaders of CHSI. The average length of general practice experience was 14.4 years, with 67.9% experts working for over 10 years in this field. There were 78.6% experts had master or PhD degree and 64.3% experts were with senior grade title (Table 1).

**Table 1:** Panel characteristics of the Delphi process (*n* = 28)

Characteristics	Frequency	Percentage (%)
Gender		
Male	10	35.7
Female	18	64.3
Age, years		
30–39	4	14.3
40–49	15	53.6
≥ 50	9	32.1
Professional field		
GPs in CHSI	9	32.1
GPs in hospital	11	39.3
Professors in medical university	3	10.7
Leader of CHSI	5	17.9
Working years		
< 10	9	32.1
≥ 10	19	67.9
Highest degree		
Bachelor	6	21.4
Master	17	60.7
PhD	5	17.9
Professional title^a^		
Middle grade title	4	14.3
Associate senior grade title	6	21.4
Senior grade title	18	64.3

^a^Note: medical professional titles include junior grade, middle grade, associate senior grade and senior grade titles, which are based upon work experience and research achievement of health professional

*Abbreviations*: *GP* General practitioner; *CHSI* Community health service institution

### First round

In the first round, 44 (95.7%) of the original 46 competencies achieved consensus in round one. The median score of importance and feasibility ranged from 8.00 to 9.00 and 7.00 to 9.00, respectively. The percentage of panel ratings in the top tertile (7–9) for importance and feasibility ranged from 85.7 to 100% and 69.9 to 100%, respectively. There were 2 competencies did not achieve 75.0% agreement in terms of feasibility, which were “3.2 Screen of at risk individuals for population health issues” (69.9% agreement) and “5.5 Allocate external resources of the institution for optimal patient care” (71.4% agreement). The indicator “3.4 Provide chemoprevention care” was recommended to be removed by 2 experts (a GP and a researcher), despite the agreement being achieved. So, three competencies were deleted in the first round.

Description of two domains were modified. “2. Basic Medical Services” was modified into “2. Patient care”. “3. Preventive care and basic public health service” was modified into “3. Basic public health service”. Eight competencies (1.10, 2.3, 2.7, 2.8, 3.7, 4.1, 4.5, and 5.4) were modified based on the experts’ comments. For example, “1.10 Record electronic health information” was modified into “1.10 Use electronic health record system effectively”. New competencies were suggested by 13 of the 28 experts in the first round. Only 7 new competencies were suggested by more than two experts and hence included in the second round (Table 2). Thus, 50 competencies were included in the second round.

**Table 2:** Results of the Delphi process

Competencies	Round 1				Round 2				Status
	Importance		Feasibility		Importance		Feasibility		
	Median	Agreement (7–9)	Median	Agreement(7–9)	Median	Agreement(7–9)	Median	Agreement(7–9)	
**1. Knowledge and skills**									
1.1 Maintain in-depth knowledge of clinical medicine	9	100%	9	100%	9	100%	9	96.4%	Included
1.2 Maintain in-depth knowledge of general practice	9	100%	9	96.4%	9	100%	9	100%	Included
1.3 Maintain in-depth knowledge of public health	9	100%	9	96.4%	9	96.4%	9	92.9%	Included
^c^1.4 Maintain in-depth knowledge of rehabilitation	-	-	-	-	8	92.9%	8	85.7%	Included
^c^1.5 Maintain in-depth knowledge of psychology and sociology	-	-	-	-	8.5	96.4%	8	89.3%	Included
1.6 Be equipped with the skill of history taking	9	100%	9	92.9%	9	96.4%	9	96.4%	Included
1.7 Be equipped with the skill of physical examination	9	100%	9	96.4%	9	100%	9	100%	Included
1.8 Interpret basic clinical tests and images correctly	9	100%	9	96.4%	9	100%	9	100%	Included
1.9 Be equipped with the skill of clinical operation	9	100%	9	96.4%	9	100%	9	100%	Included
1.10 Record electronic health information (^d^Use electronic health record system effectively)	9	100%	9	92.9%	9	100%	9	100%	Included
**2. Basic Medical Services (**^**d**^**Patient care)**									
2.1 Manage diseases at early stage presenting in an undifferentiated way	9	100%	9	96.4%	9	100%	9	96.4%	Included
2.2 Manage simultaneously multiple complaints and pathologies, both acute and chronic health problems in the individual	9	100%	9	100%	9	100%	9	100%	Included
2.3 Treat patients at acute, severe and dangerous stages (^d^Manage emergency conditions)	9	100%	9	100%	9	100%	9	100%	Included
2.4 Arrange referrals to specialists when necessary	9	100%	9	92.9%	9	96.4%	9	92.9%	Included
^c^2.5 Ensure patient safety	-	-	-	-	9	100%	9	92.9%	Included
^c^2.6 Provide advice of rehabilitation when necessary	-	-	-	-	8	100%	8	92.9%	Included
2.7 Sign contracts with patients (^d^Sign contracts with patients and provide continuous service)	9	96.4%	8.5	85.7%	9	96.4%	9	92.9%	Included
2.8 Follow-up in patients’ home (^d^Provide home visit and follow-up)	9	96.4%	8	78.6%	8.5	96.4%	8	85.7%	Included
2.9 Provide home care when necessary	9	96.4%	9	92.9%	8	89.3%	8	89.3%	Included
**3. Preventive care and basic public health service (**^d^**Basic public health service)**									
3.1 Organize health education	9	100%	9	89.3%	9	100%	9	96.4%	Included
^a^3.2 Screen of at risk individuals for population health issues	8.5	92.9%	8	69.9%	-	-	-	-	Deleted
3.3 Provide preventive care by vaccination	9	92.9%	9	92.9%	9	92.9%	9	92.9%	Included
^b^3.4 Provide chemoprevention care	8	85.7%	8	78.6%	-	-	-	-	Deleted
3.5 Establish and manage the health files	9	100%	9	92.9%	9	100%	9	100%	Included
3.6 Manage the care of special population in the community (elderly, women, children, disabled, patients with mental illness)	9	100%	8	89.3%	9	92.9%	9	96.4%	Included
3.7 Manage chronic condition (^d^Undertake the continuing management of chronic health problems)	9	100%	9	96.4%	9	100%	9	100%	Included
3.8 Identify and manage public health emergencies	9	100%	9	96.4%	9	100%	9	100%	Included
**4. Communication**									
4.1 Listen respectfully to patient and family (^d^Listen carefully to patients and be empathy)	9	100%	9	92.9%	9	100%	9	92.9%	Included
4.2 Explain things clearly and check for patients and families understanding	9	100%	9	92.9%	9	100%	8.5	92.9%	Included
4.3 Discuss with patients and families about their health condition and thoughts	9	100%	8.5	92.9%	9	96.4%	8	85.7%	Included
4.4 Propose treatment plan to patients and families	9	100%	9	100%	9	100%	8.5	92.9%	Included
4.5 Engage patient and family in making decision of therapy plan (^d^Engage patients and families in making decision of therapy plan that reflect the their needs, value and goals)	9	96.4%	9	89.3%	9	92.9%	8	85.7%	Included
4.6 Communicate effectively with colleagues	9	96.4%	9	96.4%	9	96.4%	8	89.3%	Included
4.7 Communicate effectively with stuffs in other institutions	9	96.4%	8	85.7%	8	85.7%	7	82.1%	Included
**5. Teamwork**									
5.1 Collaborate with the members in GP team	9	100%	9	96.4%	9	96.4%	8	92.9%	Included
5.2 Collaborate with other colleagues	9	100%	8	89.3%	8	92.9%	8	89.3%	Included
5.3 Collaborate with stuffs in other institutions	9	100%	8	78.6%	8	92.9%	7	82.1%	Included
5.4 Allocate internal resources of the institution for optimal patient care (^d^Allocate resources of the institution for optimal patient care)	8	96.4%	8	82.1%	8	89.3%	7	89.3%	Included
^a^5.5 Allocate external resources of the institution for optimal patient care	8	92.9%	7	71.4%	-	-	-	-	Deleted
^c^5.6 Encourage community personnel and social resources to help with community health services	-	-	-	-	9	96.4%	8	89.3%	Included
^c^5.7 Mobilize community members and social resources to provide community health services	-	-	-	-	7.5	89.3%	7	82.1%	Included
**6. Professionalism**									
6.1 Adhere to the medical rules and regulations strictly	9	100%	9	96.4%	9	100%	9	92.9%	Included
^c^6.2 Demonstrate a commitment to patients through clinical excellence and high ethical standards	-	-	-	-	9	100%	9	89.3%	Included
6.3 Adhere to patients’ right to know	9	100%	9	96.4%	9	96.4%	9	92.9%	Included
6.4 Adhere to confidentiality and privacy principles	9	100%	9	100%	9	96.4%	9	96.4%	Included
6.5 Have the sense of responsibility	9	100%	8	92.9%	9	100%	8	89.3%	Included
6.6 Self-adjust in the face of challenges	9	100%	8	78.6%	9	100%	8	89.3%	Included
**7. Education, learning and research**									
7.1 Teach students	9	96.4%	8	89.3%	8	92.9%	8	92.9%	Included
7.2 Be engaged in practice-based learning and development	9	100%	8	78.6%	9	100%	8	89.3%	Included
7.3 Be engaged in the continuous enhancement of their professional activities through ongoing learning	9	100%	8.5	96.4%	9	100%	9	100%	Included
7.4 Demonstrate an understanding of the scientific principles of research	8	96.4%	8	89.3%	8	89.3%	7	92.9%	Included
7.5 Search, navigate, and evaluate resources and clinical practice guidelines that are relevant to general practice	8	96.4%	8	89.3%	8	92.9%	8	82.1%	Included
7.6 Participate in or conduct researches in general practice	8	89.3%	8	89.3%	8	92.9%	7.5	85.7%	Included

Experts rated the importance and feasibility of each indicator on a 1–9 Likert scale (1 = not important/feasible and 9 = very important/feasible)

^a^Competencies deleted in the first round due to failure to achieve 75.0% agreement in terms of feasibility

^b^Competencies deleted in the first round due to experts’ recommendation

^c^ items added in the second round

(^d^) Competencies modified in the first round

### Second round

At this step, 50 competencies were evaluated, including retained, modified, and new competencies. In the second round, the median score of importance and feasibility ranged from 7.50 to 9.00 and 7.00 to 9.00, respectively. The percentage of panel ratings in the top tertile (7–9) for importance and feasibility ranged from 85.7 to 100% and 82.1 to 100%, respectively. As a result, more than 75% of the experts gave ratings in the top tertile (7–9) to 50 competencies, all of which had a median of 7 or above and a high degree of consensus was achieved in terms of importance and feasibility. Descriptive statistics including the median and percentage agreement for each indicator is shown in Table 2.

At the end of the Delphi process, 50 competencies finally achieved consensus in the second round in 7 domains: knowledge and skills (10 competencies), patient care (9 competencies), basic public health services (6 competencies), communication (7 competencies), teamwork (6 competencies), professionalism (6 competencies) and education, learning and research (6 competencies) (Table 3).

**Table 3:** Final competencies in the competency model for general practitioners in China

Domains	Competencies
1. Knowledge and skills	1.1 Maintain in-depth knowledge of clinical medicine
	1.2 Maintain in-depth knowledge of general practice
	1.3 Maintain in-depth knowledge of public health
	1.4 Maintain in-depth knowledge of rehabilitation
	1.5 Maintain in-depth knowledge of psychology and sociology
	1.6 Be equipped with the skill of history taking
	1.7 Be equipped with the skill of physical examination
	1.8 Interpret basic clinical tests and images correctly
	1.9 Be equipped with the skill of clinical operation
	1.10 Use electronic health record system effectively
2. Patient care	2.1 Manage diseases at early stage presenting in an undifferentiated way
	2.2 Manage simultaneously multiple complaints, both acute and chronic health problems in the individual
	2.3 Manage emergency conditions
	2.4 Arrange referrals to specialists when necessary
	2.5 Ensure patient safety
	2.6 Provide advice of rehabilitation when necessary
	2.7 Sign contracts with patients and provide continuous service
	2.8 Provide home visit and follow-up
	2.9 Provide home care when necessary
3. Basic public health services	3.1 Organize health education
	3.2 Provide preventive care by vaccination
	3.3 Establish and manage the health files
	3.4 Manage the care of special population in the community (elderly, women, children, disabled, patients with mental illness)
	3.5 Undertake the continuing management of chronic health problems
	3.6 Identify and manage public health emergencies
4. Communication	4.1 Listen carefully to patients and be empathy
	4.2 Explain things clearly and check for patients and families understanding
	4.3 Discuss with patients and families about their health condition and thoughts
	4.4 Propose treatment plan to patients and families
	4.5 Engage patients and families in making decision of therapy plan that reflect the their needs, value and goals
	4.6 Communicate effectively with colleagues
	4.7 Communicate effectively with stuffs in other institutions
5. Teamwork	5.1 Collaborate with the members in GP team
	5.2 Collaborate with other colleagues
	5.3 Collaborate with stuffs in other institutions
	5.4 Allocate resources of the institution for optimal patient care
	5.5 Demonstrate collaborative leadership in professional practice to enhance health care
	5.6 Encourage community personnel and social resources to help with community health services
6. Professionalism	6.1 Adhere to the medical rules and regulations strictly
	6.2 Demonstrate a commitment to patients through clinical excellence and high ethical standards
	6.3 Adhere to patients’ right to know
	6.4 Adhere to confidentiality and privacy principles
	6.5 Have the sense of responsibility
	6.6 Self-adjust in the face of challenges
7. Education, learning and research	7.1 Teach students
	7.2 Be engaged in practice-based learning and development
	7.3 Be engaged in the continuous enhancement of their professional activities through ongoing learning
	7.4 Demonstrate an understanding of the scientific principles of research
	7.5 Search, navigate, and evaluate resources and clinical practice guidelines that are relevant to general practice
	7.6 Participate in or conduct researches in general practice

## Discussion

### Main finding

This study was a rigorous process, which involved a multi-method approach to analyze behaviors associated with the performance of GP, including literature review, behavioral observation of GP–patient consultation, and critical incidents interview of GP. Then a modified Delphi survey was conducted with 28 general practice experts to achieve consensus on the most essential competencies of GPs after standardized residency training in China. The final consensus set included 50 competencies categorized into 7 domains.

As described in foreign competency models, the competency of GP involved many aspects, such as patient care, communication, professional knowledge and skills, professionalism and practice-based learning [14–18], which were also important for GPs’ role in China. As the first contact for patients within PHC system, the main task of GP is patient care in clinics, including medical care to patients with acute and chronic health problems. Professional knowledge and skills are the foundation of patient care. Effective communication was crucial to doctor-patient relationship [32], which was also indicated by the GPs in critical incidents interview in our study. For general practice, communication and empathy are essential in patient-centered care [33]. In addition, practice-based learning plays a very important role in the improvement of GPs ability, as the evidence or knowledge are clinically relevant and reflect the circumstances of real practice [34]. In the competency model for GPs after standardized residency training in China, the competencies mentioned above were identified as 34 items based on literature, GPs’ work content in China, and the experts’ consensus.

It is notable that there are three aspects special in China: basic public health service, teamwork, and research. To tackle health inequity, providing universal basic public health services for residents is the main goal of the new health reform in China. Since 2009, basic public health service programs have been widely carried out across PHC sectors in China [35], which now includes 14 categories, such as health records management for residents, health education, vaccination, reporting of infectious diseases and public health emergencies, and etc. [36]. GPs in PHC system play key roles in delivering majority of the basic public health services. Therefore, we made an attempt to identify competencies in this domain on the basis of basic public health services programs and the experts’ consensus. Six competencies were identified in an independent domain “3. Basic public health service”. Since 2011, many provinces and cities in China have explored the model of “family doctor contract” services, which help to let patients have their personal doctors and improve the continuity of care based on GP team with a GP, a nurse, and a preventive care physician [1, 37]. The ability of working effectively with others in a collaborative team-based model is emphasized to GPs in China. Besides, collaborative leadership is also an important indicator as GP plays the role of leader in the team. In China, research ability and paper are critical to physicians in career advancement. During the standardized training program, GPs can be trained with the ability of scientific research [11], which should be evaluated. Therefore, three domains of competencies were included in our competency model.

Compared with previous assessment tools of GPs’ competency in China, the competency model for GPs after standardized residency training in this study made improvement on method and content. A literature review of 31 studies evaluating competencies of GPs in China, conducted by our research team before this research, found that more than half of the included studies did not use a psychometrically robust, high-quality instrument to measure the competency of GP [20]. In this study, the competency model for GPs after standardized residency training was developed through a modified Delphi method. The Delphi questionnaire encompassed previous studies regarding GP competencies and published competency models from international general practice organizations [14–18]. In addition, further competencies related to the work content of GPs were added by behavioral observation of GP–patient consultations and critical incidents interview of GPs [25]. These two methods had not been introduced in the process of developing competency assessment tools for Chinese GP before. Besides, for the “5 + 3” residency training model, the evaluation of GP trainees usually focused on process assessment to monitor the progress of training. In this study, we developed a competency model for evaluation of GPs after standardized residency training. In questionnaire preparation, the Content and Rules for Standardized Training of General Practitioners (2019 revised edition) was referred [11]. In Delphi process, the experts were familiar with “5 + 3” residency training. Therefore, this competency model can be used to explore the performance and competency of GPs after standardized residency training in workplace. The results may provide feedback to GPs and trainers of the “5 + 3” residency training model for further improvement.

In this study, the importance and feasibility of the competencies achieved consensus in the Delphi process. This set of competencies provided a basis for competency measurement of GPs after standardized residency training in China, which still needs to be tested in practice in further studies [38]. We suggest compiling these competencies in our model into a questionnaire for self-evaluation by GPs or multi-source assessment by other staff and patients in workplace [39].

### Strengths and limitations

The quality of panel experts and their opinions on given topics is seen as key factor of the Delphi technique [40]. In this study, the presence of different professionals (GPs in hospitals, GPs in CHSIs, leaders of CHSIs, and researchers in universities) and geographical areas (28 experts from 7 provinces of China), along with the average length of general practice experience (14.4 years) and similar ratio of experts with GPs in actual practice (64.3% female experts VS 58.0% female GPs in actual practice [6]), suggested that our expert panel represented a broad and experienced group. Furthermore, the response rate of our study was 100% in two rounds of Delphi process. This was a satisfactory result as response rate was a recognized problem in Delphi study. Importantly, this meant that experts had strong interest and active participation in this topic.

There are limitations of this study. First, although experts in this study were from different geographical areas, most of them were from Beijing and the proportion of experts in other provinces was low. They may not adequately represent the full spectrum of views held by individuals in different regions across China. Second, patients’ opinion was not involved in the sources of competency. Patients are the customers and beneficiaries of general practice services, who may give deep insights in health care experience and doctor-patient communication [25]. This should be taken into consideration in further study. The methodology of Delphi process relies on the perception of experts, which may entail further evidence from implementation in real practice settings [41]. Further study is needed to apply this competency model and confirm the validity of these competencies.

## Conclusion

Based on a systematic consensus process, the competency model for GPs after standardized residency training in China has been developed and described. This model can be used in self-evaluation and multi-source feedback to explore GPs’ clinical performance and professional behaviour. Before application in general practice, this competency model still need to be validated in a further study.

## Supplementary Information

**Additional file 1.** References of the 37 papers by literature review.

## Data Availability

The data used and/or analyzed during the current study are available from the corresponding author on reasonable request.

## Abbreviations

PHC: Primary health care
GPs: General practitioners
WONCA: The World Organization of Family Doctors
CFPC: The College of Family Physicians of Canada
ACGME: The Accreditation Council for Graduate Medical Education
RCGP: The Royal College of General Practitioners
RACGP: The Royal Australian College of General Practitioners
CHSI: Community health service institution
RFEs: Reasons for encounter
