# Supplementary material for: Development of a competency model for general practitioners after standardized residency training in China by a modified Delphi method
Source: BMC Fam Pract. 2021 Aug 26;22:171. doi: 10.1186/s12875-021-01508-7 (PMC8390270; doi:10.1186/s12875-021-01508-7)
Supplement: Supplementary file 1 — Additional file 1. References of the 37 papers by literature review. [file 12875_2021_1508_MOESM1_ESM.docx]

Supplementary file 1. References of the 37 papers by literature review

**References**

1. DU Gai-yan, QI Dian-jun, WANG Shuang, ey al. Exploration and Practice of the Residency Training Implementation Plan in General Practice [in Chinese]. Chinese General Practice, 2011, 14(10):1109-1111. DOI:10.3969/j.issn.1007-9572.2011.10.019.
2. MA Zhi-qiang, LIU Min, WANG Hai-rong. Empirical research on competency of general practitioners in community health service institutions [in Chinese]. Journal of Shanghai Jiaotong University(Medical Science),2012,32(4):514-518. DOI:10.3969/j.issn.1674-8115.2012.04.030.
3. PAN Xiao-yan.Evaluation index system of general practitioner quality in Guangxi [in Chinese]. Hunan:Central South University,2013.
4. JIN Li-jiao, JIA Ying-lei, SUN Tao, et al. Competency Model of General Practitioners: An Exploratory Factor Analysis [in Chinese]. Chinese General Practice. 2013,16(11):3659-3661. DOI:10.3969/j.issn.1007-9572.2013.31.006
5. [SHOU Juan](http://g.wanfangdata.com.cn/details/detail.do?_type=perio&id=zhqkyszz201301015), [ZHU Shan-zhu](http://g.wanfangdata.com.cn/details/detail.do?_type=perio&id=zhqkyszz201301015), [ZHANG Xiang-jie](http://g.wanfangdata.com.cn/details/detail.do?_type=perio&id=zhqkyszz201301015), et al. Establishment of assessment system for comprehensive clinical competency of general practice residents [in Chinese].
   Chinese Journal of General Practitioners. [2013,12(1)](http://g.wanfangdata.com.cn/details/javascript:void(0)):25-29. DOI:[10.3760/cma.j.issn.1671-7368.2013.01.015](http://g.wanfangdata.com.cn/details/javascript:void(0);)
6. XU Long-long， SU Ting-ting， WANG Shuang， et al. A Preliminary Study on the Assessment Index System of Clinical Core Competencies for the Community General Practitioners [in Chinese]. Chinese General Practice. 2013,13(10):3358-3361. DOI:[10.3969/j.issn.1007-9572.2013.28.022](http://g.wanfangdata.com.cn/details/javascript:void(0);)
7. DONG Hai-na, ZHU Shun, WANG Wei-jie. Application of Fuzzy Comprehensive Evaluation Method in the Evaluation of Core Competence of General Practitioners [in Chinese]. China Health Statistics,[2014,31(2)](http://g.wanfangdata.com.cn/details/javascript:void(0)):273-274,277.
8. [YE Teng-fei](http://g.wanfangdata.com.cn/details/detail.do?_type=perio&id=zgqkyx201407007), [PAN Xiao-yan](http://g.wanfangdata.com.cn/details/detail.do?_type=perio&id=zgqkyx201407007). Construction of Evaluating Index System Based on the AHP on the Competence of Urban General Practitioner in Guangxi [in Chinese].
   Chinese General Practice. 2014,17(7):752-755．DOI:[10.3969/j.issn.1007-9572.2014.07.006](http://g.wanfangdata.com.cn/details/javascript:void(0);)
9. [ZHU Shun](http://g.wanfangdata.com.cn/details/detail.do?_type=perio&id=zgqkyx201404005), [DONG Hai-na](http://g.wanfangdata.com.cn/details/detail.do?_type=perio&id=zgqkyx201404005), [WANG Wei-jie](http://g.wanfangdata.com.cn/details/detail.do?_type=perio&id=zgqkyx201404005). Construction of Core Competency Evaluation Index System for General Practitioner [in Chinese]. Chinese General Practice.2014,17(4):378-380. DOI:[10.3969/j.issn.1007-9572.2014.04.005](http://g.wanfangdata.com.cn/details/javascript:void(0);)
10. [SHEN Qun-hong](http://g.wanfangdata.com.cn/details/detail.do?_type=perio&id=zgwszy201506016), [WANG Zheng](http://g.wanfangdata.com.cn/details/detail.do?_type=perio&id=zgwszy201506016), [WANG Pan-pan](http://g.wanfangdata.com.cn/details/detail.do?_type=perio&id=zgwszy201506016), et al. Research on building the model of community general practitioners’ competencies [in Chinese]. [Chinese Health Resources](http://g.wanfangdata.com.cn/details/javascript:void(0)). [2015,(6)](http://g.wanfangdata.com.cn/details/javascript:void(0)):411-415. DOI:[10.13688/j.cnki.chr.2015.15174](http://g.wanfangdata.com.cn/details/javascript:void(0);)
11. SU Fang, ZUO Yan-li, WU Cai-yuan, et al. Establishment of Competency Model for General Practitioners in Guangxi Township Health Centers [in Chinese]. Chinese General Practice.[2015,(16)](http://g.wanfangdata.com.cn/details/javascript:void(0)):1893-1900. DOI:[10.3969/j.issn.1007-9572.2015.16.007](http://g.wanfangdata.com.cn/details/javascript:void(0);)
12. WU Zhen-zhen. Research on building and applying the competency model of family doctor -a case study of Q district in Shanghai [in Chinese]. Shanghai:Donghua University, 2016.
13. WANG jing. Establishing and Empirical Study on Post Competency Model for General Practitioner. Shanxi:Shanxi Medical University,2016
14. [ZHU Wen-hua](http://g.wanfangdata.com.cn/details/detail.do?_type=perio&id=zgqkyx201634018), [FANG Li-zheng](http://g.wanfangdata.com.cn/details/detail.do?_type=perio&id=zgqkyx201634018), [WANG Xiao-jing](http://g.wanfangdata.com.cn/details/detail.do?_type=perio&id=zgqkyx201634018), et al. Exploration of Multidimensional Capability Assessment of General Practice Residency Based on Post Competency [in Chinese]. Chinese General Practice. 2016,19(34):4220 -4224.DOI:10.3969/j.issn.1007-9572.2016.34.015
15. [SHEN Zheng-fu](http://g.wanfangdata.com.cn/details/detail.do?_type=perio&id=bbyxyxb201601029), [LI Dan-dan](http://g.wanfangdata.com.cn/details/detail.do?_type=perio&id=bbyxyxb201601029), [QI Yu-long](http://g.wanfangdata.com.cn/details/detail.do?_type=perio&id=bbyxyxb201601029), et al. A study on the competency evaluation index system of the rural general practitioners [in Chinese]. Journal of Bengbu Medical College. [2016,41(1)](http://g.wanfangdata.com.cn/details/javascript:void(0)):84-88. DOI:[10.13898/j.cnki.issn.1000-2200.2016.01.027](http://g.wanfangdata.com.cn/details/javascript:void(0);)
16. WANG Fang.Evaluation index system of general practitioner quality.Shandong:Qingdao University,2017.
17. [LIU Hong](http://g.wanfangdata.com.cn/details/detail.do?_type=perio&id=zgqkyx201725007), [SHI Wei-hong](http://g.wanfangdata.com.cn/details/detail.do?_type=perio&id=zgqkyx201725007), [QIAO Xue-bin](http://g.wanfangdata.com.cn/details/detail.do?_type=perio&id=zgqkyx201725007). Competencies of Grassroots General Practitioners in Jiangsu Province [in Chinese]. Chinese General Practice. 2017，20(25)：3088-3093.DOI:[10.3969/j.issn.1007-9572.2017.25.006](http://g.wanfangdata.com.cn/details/javascript:void(0);)
18. [HAN Ying](http://g.wanfangdata.com.cn/details/detail.do?_type=perio&id=zgqkyx201701006), [WANG Jing](http://g.wanfangdata.com.cn/details/detail.do?_type=perio&id=zgqkyx201701006), [ZHENG Jian-zhong](http://g.wanfangdata.com.cn/details/detail.do?_type=perio&id=zgqkyx201701006), et al. Construction of Evaluation Index System of Post Competency of General Practitioners [in Chinese]. Chinese General Practice. 2017,20(1):15-20. DOI:[10.3969/j.issn.1007-9572.2017.01.004](http://g.wanfangdata.com.cn/details/javascript:void(0);)
19. WU Wen-jun, YE Zi-hui,SUN jin-ming,et al. Research on competency and training strategy of general practice students under the background of smart medical care [in Chinese]. [Zhejiang Medical Journal](http://www.wanfangdata.com.cn/perio/detail.do?perio_id=zjyx&perio_title=Zhejiang Medical Journal" \t "http://d.wanfangdata.com.cn/periodical/_blank),2017,39(7):592-594.DOI:10.12056/j.issn.1006-2785.2017.39.7.2016-2097
20. HUANG FU Hui-hui, LI Hong-yan.Evaluation Model and Empirical Study of Family Doctor Competency in Shanghai Based on Meta-competence [in Chinese]. Modern Hospital Management,2018,16(3):4-8.DOI:10.3969/j.issn.1672-4232.2018.03.002.
21. WANG Meei-rong, GE Cai-ying, ZHANG Dan-dan, et al. Evaluation Index System Construction of Consultation Competence of Training of General Practitioners [in Chinese]. Medicine and Society,2018,31(4):70-73.DOI:10.13723/j.yxysh.2018.04.022.
22. YIN Zhu-ping, LIANG Hong-min, PENG Yun-zhu, et al. Developing a general practitioners' professional capacities model in the ethnic minority region of Yunnan Province [in Chinese]. China Higher Medical Education, 2018,(9):9-10,30.DOI:10.3969/j.issn.1002-1701.2018.09.005.
23. LI Mi-qiong, WU Yu-miao, ZHU Jie, et al. Development of a Family Doctor Performance Evaluation System Using the Delphi Method [in Chinese]. Chinese General Practice, 2019,22(16):1989-1995. DOI:10.12114/j.issn.1007-9572.2019.00.193.
24. ZHU Li. The formulation of the theoretical model and evaluation system for the competency of family doctors [in Chinese]. Healthmust-Readmagazine, 2019,(28):267-268.
25. LUO Xiao-lu, HUANG Yan-li, HAO Jia-ping,et al. Development of a Service Capability Building Evaluation System for Family Doctor Teams [in Chinese]. Chinese General Practice, 2019,22(13):1554-1558.DOI:10.12114/j.issn.1007-9572.2019.00.216.
26. SHANG Xiao-peng, YANG Qing, QIU Yin-wei, et al. Development of a Contracted Service Performance Evaluation System for Family Doctor Teams [in Chinese]. Chinese General Practice, 2019,22(16):1996-1999,2007.DOI:10.12114/j.issn.1007-9572.2018.00.386.
27. LU Zhi-min, LU Ping. Exploratory Analysis of Competency Indicators for General Practitioners [in Chinese]. Chinese General Practice, 2019,22(28):3495-3500. DOI:10.12114/j.issn.1007-9572.2019.00.389.
28. LU Ping, LU Zhi-min, QIAN Zhi-fang. Construction of Family Doctor's Competency Indicator System Based on the Delphi Method [in Chinese]. Chinese General Practice,2020,23(28):3553-3560.DOI:10.12114/j.issn.1007-9572.2020.00.069.
29. SHAO Qian, WU Yan, WANG Zi-jing, et al. Building of a competency model of family doctor team: A survey in Anhui Province [in Chinese]. Chinese Rural Health Service Administration,2020,40(6):393-398.
30. Zou Hai-yan, Gao Hong-xia, Chen Meng-xue, et al. Theoretical construction of competency model for rural family physicians on contract [in Chinese]. Chinese Journal of Hospital Administration, 2020,36(5):417-421.DOI:10.3760/cma.j.cn111325-20200215-00151.
31. FANG Jin-ming, TAO Hong-bin, PENG Yi-xiang, et al. Construction of Post Competency Model for General Practitioners [in Chinese]. Medicine and Society,2020,33(1):129-133. DOI:10.13723/j.yxysh.2020.01.030.
32. [Patterson F](https://www.ncbi.nlm.nih.gov/pubmed/?term=Patterson F[Author]&cauthor=true&cauthor_uid=10750226), [Ferguson E](https://www.ncbi.nlm.nih.gov/pubmed/?term=Ferguson E[Author]&cauthor=true&cauthor_uid=10750226), [Lane P](https://www.ncbi.nlm.nih.gov/pubmed/?term=Lane P[Author]&cauthor=true&cauthor_uid=10750226), et al. A competency model for general practice: implications for selection, training, and development. British Journal of General Practice. 2000,50(452):188-93.
33. [van de Camp K](https://www.ncbi.nlm.nih.gov/pubmed/?term=van de Camp K[Author]&cauthor=true&cauthor_uid=16441322), [Vernooij-Dassen M](https://www.ncbi.nlm.nih.gov/pubmed/?term=Vernooij-Dassen M[Author]&cauthor=true&cauthor_uid=16441322), [Grol R](https://www.ncbi.nlm.nih.gov/pubmed/?term=Grol R[Author]&cauthor=true&cauthor_uid=16441322), et al. Professionalism in general practice_ development of an instrument to assess professional behavior in general practitioner trainees. Medical Education. 2006,40(1):43-50. DOI:[10.1111/j.1365-2929.2005.02346.x](https://doi.org/10.1111/j.1365-2929.2005.02346.x)
34. [Allen T](https://www.ncbi.nlm.nih.gov/pubmed/?term=Allen T[Author]&cauthor=true&cauthor_uid=21918130), [Brailovsky C](https://www.ncbi.nlm.nih.gov/pubmed/?term=Brailovsky C[Author]&cauthor=true&cauthor_uid=21918130), [Rainsberry P](https://www.ncbi.nlm.nih.gov/pubmed/?term=Rainsberry P[Author]&cauthor=true&cauthor_uid=21918130), et al. Defining competency-based evaluation objectives in family medicine_ dimensions of competence and priority topics for assessment Canadian Family Physician. 2011,57(9):e331-40.
35. [Longenecker RL](https://www.ncbi.nlm.nih.gov/pubmed/?term=Longenecker RL[Author]&cauthor=true&cauthor_uid=29346700), [Wendling A](https://www.ncbi.nlm.nih.gov/pubmed/?term=Wendling A[Author]&cauthor=true&cauthor_uid=29346700), [Hollander-Rodriguez J](https://www.ncbi.nlm.nih.gov/pubmed/?term=Hollander-Rodriguez J[Author]&cauthor=true&cauthor_uid=29346700), et al. Competence Revisited in a Rural Context. Family Medicine, 2018,50(1):28-36. DOI: 10.22454/FamMed.2018.712527.
36. [Mitsuyama T](https://www.ncbi.nlm.nih.gov/pubmed/?term=Mitsuyama T[Author]&cauthor=true&cauthor_uid=30497398), [Son D](https://www.ncbi.nlm.nih.gov/pubmed/?term=Son D[Author]&cauthor=true&cauthor_uid=30497398), [Eto M](https://www.ncbi.nlm.nih.gov/pubmed/?term=Eto M[Author]&cauthor=true&cauthor_uid=30497398). Competencies required for general practitioners/family physicians in urban areas versus non-urban areas_ a preliminary study. BMC Family Practice. 2018,29;19(1):186. DOI: 10.1186/s12875-018-0869-4.
37. [Covert H](https://www.ncbi.nlm.nih.gov/pubmed/?term=Covert H[Author]&cauthor=true&cauthor_uid=30571307), [Sherman M](https://www.ncbi.nlm.nih.gov/pubmed/?term=Sherman M[Author]&cauthor=true&cauthor_uid=30571307), [Miner K](https://www.ncbi.nlm.nih.gov/pubmed/?term=Miner K[Author]&cauthor=true&cauthor_uid=30571307), et al. Core Competencies and a Workforce Framework for Community Health Workers: A Model for Advancing the Profession. The American Journal of Public Health. 2019,109(2):320-327. DOI:10.2105/AJPH.2018.304737. Epub 2018 Dec 20.
